# Uremic Toxin-Producing *Bacteroides* Species Prevail in the Gut Microbiota of Taiwanese CKD Patients: An Analysis Using the New Taiwan Microbiome Baseline

**DOI:** 10.3389/fcimb.2022.726256

**Published:** 2022-04-26

**Authors:** Subhashree Shivani, Cheng-Yen Kao, Amrita Chattopadhyay, Jenn-Wei Chen, Liang-Chuan Lai, Wei-Hung Lin, Tzu-Pin Lu, I-Hsiu Huang, Mong-Hsun Tsai, Ching-Hao Teng, Jiunn-Jong Wu, Yi-Hsien Hsieh, Ming-Cheng Wang, Eric Y. Chuang

**Affiliations:** ^1^ Graduate Institute of Biomedical Electronics and Bioinformatics, National Taiwan University, Taipei, Taiwan; ^2^ Institute of Microbiology and Immunology, College of Life Sciences, National Yang Ming Chiao Tung University, Taipei, Taiwan; ^3^ Center for Translational Genomic Research, Department of Medical Research, China Medical University Hospital, Taichung, Taiwan; ^4^ Department of Microbiology and Immunology, College of Medicine, National Cheng Kung University, Tainan, Taiwan; ^5^ Graduate Institute of Physiology, College of Medicine, National Taiwan University, Taipei, Taiwan; ^6^ Bioinformatics and Biostatistics Core, Center of Genomic and Precision Medicine, National Taiwan University, Taipei, Taiwan; ^7^ Department of Internal Medicine, National Cheng Kung University Hospital, College of Medicine, National Cheng Kung University, Tainan, Taiwan; ^8^ Department of Public Health, Institute of Epidemiology and Preventive Medicine, National Taiwan University, Taipei, Taiwan; ^9^ Department of Biochemistry and Microbiology, Oklahoma State University Center for Health Sciences, Tulsa, OK, United States; ^10^ Institute of Biotechnology, National Taiwan University, Taipei, Taiwan; ^11^ Institute of Molecular Medicine, College of Medicine, National Cheng Kung University, Tainan, Taiwan; ^12^ Department of Biotechnology and Laboratory Science in Medicine, School of Biomedical Science and Engineering, National Yang-Ming Chiao Tung University, Taipei, Taiwan; ^13^ Department of Biochemistry, School of Medicine, Chung Shan Medical University, Taichung, Taiwan; ^14^ Master Program for Biomedical Engineering, China Medical University, Taichung, Taiwan

**Keywords:** gut microbiota, Taiwan, chronic kidney disease, dysbiosis, hemodialysis, peritoneal dialysis, uremic toxin

## Abstract

**Rationale and Objective:**

Gut microbiota have been targeted by alternative therapies for non-communicable diseases. We examined the gut microbiota of a healthy Taiwanese population, identified various bacterial drivers in different demographics, and compared them with dialysis patients to associate kidney disease progression with changes in gut microbiota.

**Study Design:**

This was a cross-sectional cohort study.

**Settings and Participants:**

Fecal samples were obtained from 119 healthy Taiwanese volunteers, and 16S rRNA sequencing was done on the V3-V4 regions to identify the bacterial enterotypes. Twenty-six samples from the above cohort were compared with fecal samples from 22 peritoneal dialysis and 16 hemodialysis patients to identify species-level bacterial biomarkers in the dysbiotic gut of chronic kidney disease (CKD) patients.

**Results:**

Specific bacterial species were identified pertaining to different demographics such as gender, age, BMI, physical activity, and sleeping habits. Dialysis patients had a significant difference in gut microbiome composition compared to healthy controls. The most abundant genus identified in CKD patients was *Bacteroides*, and at the species level hemodialysis patients showed significant abundance in *B. ovatus*, *B. caccae, B. uniformis*, and peritoneal dialysis patients showed higher abundance in *Blautia producta* (p ≤ 0.05) than the control group. Pathways pertaining to the production of uremic toxins were enriched in CKD patients. The abundance of the bacterial species depended on the type of dialysis treatment.

**Conclusion:**

This study characterizes the healthy gut microbiome of a Taiwanese population in terms of various demographics. In a case-control examination, the results showed the alteration in gut microbiota in CKD patients corresponding to different dialysis treatments. Also, this study identified the bacterial species abundant in CKD patients and their possible role in complicating the patients’ condition.

**Graphical Abstract:**
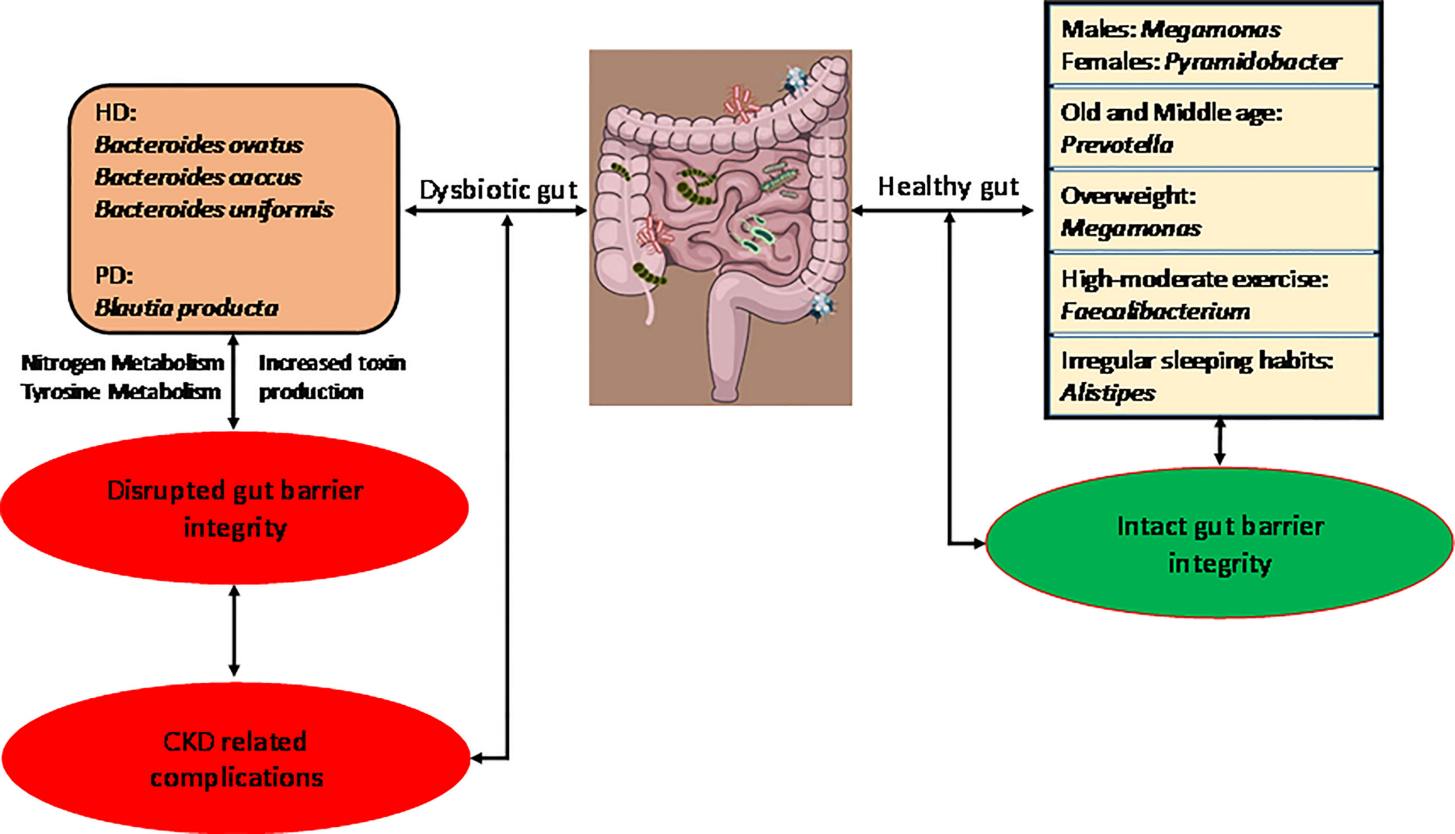
Graphical summary of our research.

## 1 Introduction

More than 100 trillion microbes cohabitate inside the human gut, influencing nutrient metabolism, immunomodulation, and antimicrobial protection of the host (Sekirov et al., 2010). Understanding the diversity in the “healthy microbiome” has been a major challenge in microbiome research. The gut microbiota harbors more than 100 bacterial species (Rowland et al., 2018). Characterizing these in healthy populations is an important initial step towards comprehending its contribution to health and disease. The Human Microbiome Project has been one such endeavor. (Turnbaugh et al., 2007) However, variability of microbiota across samples from varied ethnicities, geographical regions, and lifestyles has been observed to interfere with the composition and dynamics of the microbiome in healthy humans. (Yatsunenko et al., 2012; Senghor et al., 2018; Kim et al., 2020). Existing microbiome studies on healthy Taiwanese populations have focused on identifying enterotypes (Liang et al., 2017) and comparing the microbiome composition between normal weight and obese people (Chiu et al., 2014). However, a complete understanding of microbiome diversity related to age, gender, BMI, physical activity and sleeping habits is yet to be realized. Therefore, establishing a comprehensive reference microbiome panel characterizing the healthy gut microbiome for a Taiwanese cohort is needed.

Multiple studies have demonstrated the supremacy of the environment over host genetics in determining the intestinal microbiome composition (Rothschild et al., 2018). Researchers have amply studied the beneficial effects of the symbiotic microbe-host relationship (Kaiko and Stappenbeck, 2014); however, dysbiosis of the gut microbiota have been associated with diseases such as obesity, cancer, diabetes, inflammatory bowel disease, cardiovascular disease (CVD), and kidney disease (West et al., 2015). Chronic kidney disease (CKD) is cited as a major health issue, as patients with CKD often develop CVD, eventually succumbing to cardiovascular-associated mortality (Anavekar and Pfeffer, 2004; Hwang et al., 2010). The pathogenesis of dysbiotic gut microbiota in CKD patients has been associated with further aggravation of kidney diseases and related complications (Chen et al., 2019). Moreover, the metabolic effects of gut microbiota lead to the formation of precursors of uremic toxins in the body, which in due course contributes to cardiovascular morbidity and mortality (Castillo-Rodriguez et al., 2018). Despite this, the role of identified bacterial taxa in producing uremic toxins is largely unknown. This is a major bottleneck in developing alternative therapies for the reduction of toxins in CKD.

In this pioneering study, we first established Taiwan Microbiome Baseline (TMB), a baseline gut microbiome panel from a healthy Taiwanese cohort. We then utilized some samples from TMB as a reference control and conducted a case-control study of patients with CKD undergoing different types of dialysis, hemodialysis (HD) or peritoneal dialysis (PD). We characterized the existing differences in microbiome composition between healthy and CKD patients and identified the bacterial taxa at the species level to obtain CKD biomarkers specific to Taiwanese patients, with the goal of providing insight into the pathogenic mechanisms underlying CKD.

## 2 Materials and Methods

### 2.1 Study Participants

One hundred nineteen volunteers of Taiwanese origin were enrolled in the baseline control panel study. The healthy controls were recruited through the Health Management Center of National Taiwan University Hospital (NTUH) and underwent routine and basic health checks and physical examinations. To characterize TMB, the fecal samples were assessed by sequencing the V3-V4 regions of the 16S rRNA gene (http://twnbiome.cgm.ntu.edu.tw/). The alpha rarefaction curve reached an asymptote, suggesting sufficient sequencing depth to represent the majority of bacterial community diversity. The anthropometric data of the individuals were measured, and other demographics were evaluated based on a clinical questionnaire. Participants with autoimmune or general or gastrointestinal diseases and those using drugs/antibiotics were excluded from the study (**Table 1**). Furthermore, 16 HD and 22 PD patients from National Cheng-Kung University hospital were enrolled as CKD cases (**Table 2**). This study was approved by the Institutional Review Board of the National Taiwan University (201801085RINB) and National Cheng Kung University Hospital (B-ER-106-058). All participants provided written informed consent.

**Table 1:** Anthropometric and demographic data of healthy Taiwanese cohort.

Characteristic	Healthy group(N = 119)
Gender (male/female)	38/81
Age (mean ± SD years)	47.1 ± 15
Age (adult/middle/old)^a^	55/45/19
BMI (normal/overweight)^b^	81/38
Physical activity (high/moderate/light/sedentary)	5/54/38/22
Diet type (meat-based/half and half/plant-based/vegetarian)^c^	18/71/26/4
Sleeping habits (normal/abnormal)^d^	49/70

a Adult: 18 – 44 years, middle age: 45 – 64 years, old age: ≥ 65 years.

b Normal: 18.5 ≤ BMI < 24, overweight: 24 ≤ BMI < 27.

c Meat-based: more amount of meat than plants, half and half: similar amount of meat and plants, plant-based: more amount of plants than meat, vegetarian: only plant-based diet.

d Normal: early to bed/early to rise or late to bed/late to rise, abnormal: early to bed/late to rise or late to bed/early to rise.

**Table 2:** Anthropometric and demographic data of the dialysis patients and healthy controls.

Characteristic	Normal (n = 26)	Hemodialysis (n = 16)	Peritoneal dialysis (n = 22)	P value
Age (year)	53.6 ± 16.9	64 ± 11	51 ± 12	0.0016
Gender (Male/Female)	12/14	6/10	7/15	0.7155
Dialysis vintage (year)		5.3 ± 3.6	5.3 ± 3.4	0.9843
Diabetes mellitus		8 (50)	2 (9)	0.0047
Hypertension		14 (88)	20 (91)	0.7353
Coronary artery disease		6 (38)	1 (5)	0.0097
Stroke		0	0	—
Malignancy		2 (13)	0	0.0884
Systemic lupus erythematosus		0	1 (5)	0.5156
Constipation		10 (63)	8 (36)	0.1111
Cause of chronic kidney disease				0.0330
Diabetes		8 (50)	2 (9)	
Hypertension		3 (19)	4 (18)	
Glomerulonephritis		1 (6)	9 (41)	
Lupus nephritis		0	1 (5)	
Polycystic kidney disease		1 (6)	0	
Chronic tubulointerstitial nephritis		0	2 (9)	
Obstructive uropathy		1 (6)	0	
Others		2 (13)	4 (18)	

Data are presented with mean ± SD or number (percentage).

For the case-control analysis, 26 samples from the TMB group with normal lifestyle, no usage of supplements and comparable age and gender distribution were included. Patients undergoing HD and PD received regular diet control and renal diet education by dietician, including potassium, phosphate, purine, protein, carbohydrate, cholesterol and fat in food according to the results of monthly blood tests. However, we did not control the food intake of healthy control.

### 2.2 Sample Processing and Sequencing

#### 2.2.1 DNA Extraction

Bacterial genomic DNA was extracted from 200 mg frozen stool samples using a QIAamp Fast DNA Stool Mini kit (QIAGEN, Germany). The DNA samples (DNA yield ~1-2 μg) were stored at -20°C before PCR amplification.

#### 2.2.2 PCR Amplification and Clean-Up

First, DNA was normalized to 25 ng/μl. Forward (5’-CCTACGGGNGGCWGCAG-3’) and reverse primers (5’-GACTACHVGGGTATCTAATCC-3’) complementary to upstream and downstream sequences of the V3-V4 region of the 16S rRNA gene were designed with Illumina overhang adapters and were then used to amplify templates from bacterial genomic DNA. PCR was performed in a thermal cycler at 95°C for 3 min, followed by 25 cycles of 95°C for 30 s, annealing at 55°C for 30 s, extension at 72°C for 30 s, and a final extension at 72°C for 5 min. PCR products were then purified with a GenepHlow Gel/PCR purification kit (Geneaid, Taipei, Taiwan).

#### 2.2.3 Index PCR and Clean-Up

Illumina sequencing adapters and dual indices were attached to the PCR products using the Nextera XT Index Kit (Illumina Inc., San Diego, CA, USA). Subsequently, AMPure XP beads were used to clean up the final libraries. The expected size of the final libraries was approximately 630 bp.

#### 2.2.4 Normalization and Sequencing

Libraries were normalized and pooled, and then sequenced on the MiSeq system using v3.0 reagents (paired-end 300 bp, Illumina).

### 2.3 Data Analysis

Qiime2-2018.4 was used to process the sequenced data (Bolyen et al., 2019). Raw sequence data were de-multiplexed and sequence quality control was performed using DADA2 (Callahan et al., 2016). The feature tables were rarefied to 32,000 sequences per sample to retain the maximum observed operational taxonomic unit (OTUs, represents species richness) in the datasets. Phylogenetic trees were generated using the ‘qiime phylogeny align-to-tree-mafft-fasttree’ plugin in qiime2. The taxonomic classification of the microbial community was done using the ‘qiime feature-classifier’ plugin. Characterizations of the structure of the microbial community were done based on alpha diversity (within-sample) and beta diversity (between samples). Alpha diversity metrics included observed OTUs, Pielou’s evenness (amount of evenness relative to the maximum and minimum possible for a given richness) (Pielou, 1966). Faith’s phylogenetic diversity (community richness incorporating phylogenetic relationships between features) (Faith, 1992), and Shannon diversity (characterizes species abundance and evenness) (Shannon, 1948). Beta diversity metrics included weighted UniFrac (community dissimilarity based on phylogenetic relationships between the features) (Lozupone et al., 2011), unweighted UniFrac (qualitative measure of community dissimilarity that incorporates phylogenetic relationships between the features) (Lozupone et al., 2011) and Bray-Curtis distance (quantitative measure of community dissimilarity) (“295_Sørensen, Thorvald.Pdf”, 1948). Principal coordinate analysis (PCoA) was used to ordinate the output matrices and was visualized using EMPeror (Vázquez-Baeza et al., 2013). LEfSe (linear discriminant analysis effect size) (Segata et al., 2011) was used to identify the abundant bacterial species in different groups. Additionally, the OTUs of identified bacteria were obtained from the taxonomy table and comparison analyses were conducted among HD, PD and control groups. Fisher’s exact test (p ≤ 0.05) was used to compare the presence/absence of significantly identified bacteria between groups by analyzing the presence of the said bacterial OTUs in the sample. Bacteria with OTUs > 0 were deemed as “present” in the sample. A Wilcoxon test was performed to test the differential abundance of identified bacterial OTUs between two groups. Abundance percentage of identified bacteria was calculated for individual samples from the OTU table. Average abundance was calculated for each group and compared with other groups. Finally PICRUSt (phylogenetic investigation of communities by reconstruction of unobserved states) (Langille et al., 2013) was used to predict the functional gene content in the fecal microbiota based on taxonomy obtained from the Greengenes reference database (DeSantis et al., 2006).

## 3 Results

### 3.1 Composition of the Healthy Gut Microbiome

#### 3.1.1 Bacterial Composition

**Figure 1:**
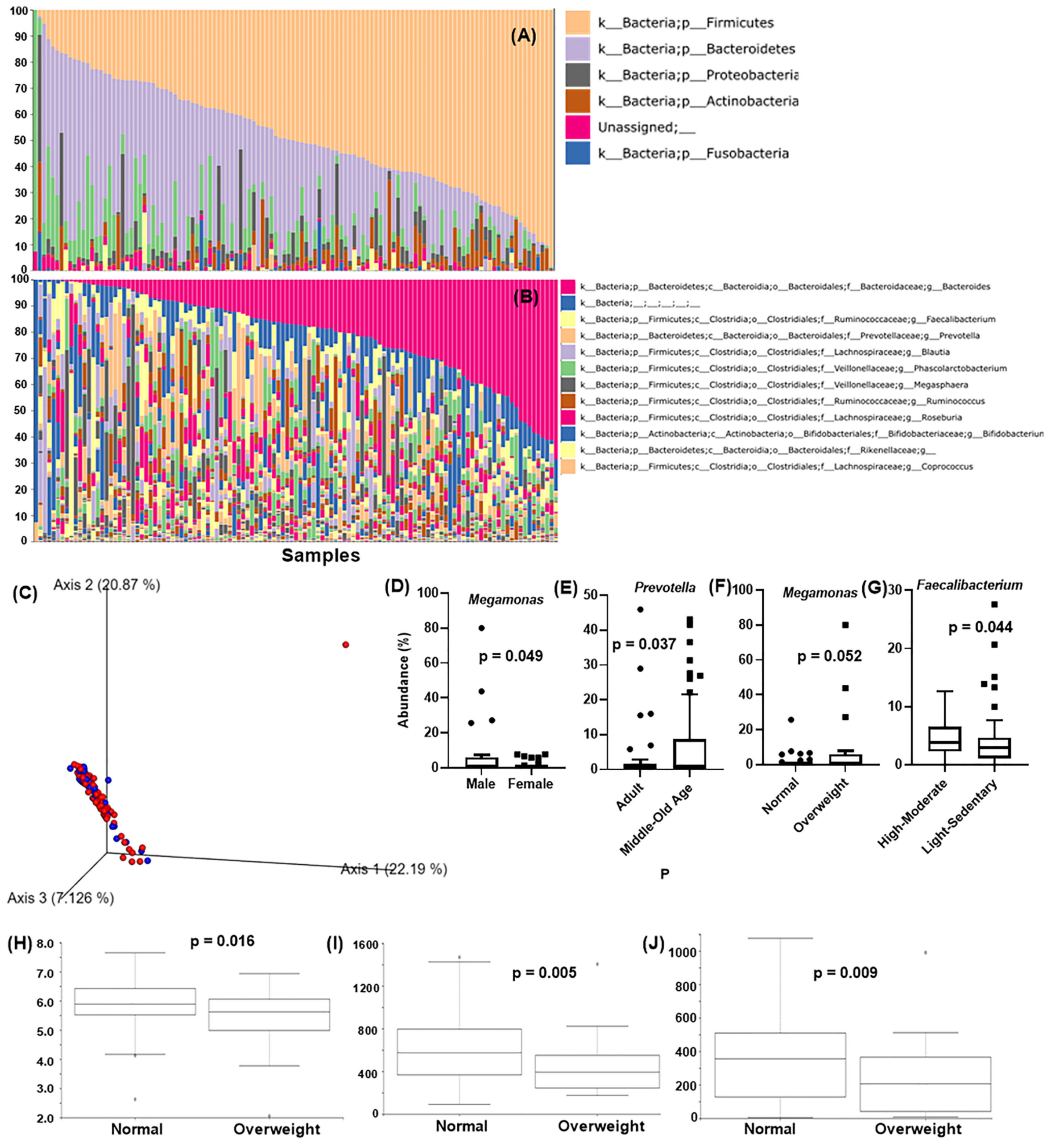
Analysis of Taiwanese Microbiome Baseline. Taxonomic classification at the **(A)** phylum level and **(B)** genus level. X-axis: individual samples (N=119), y-axis: percentage of bacteria in individual samples. **(C)** PCoA of the beta diversity values based on weighted UniFrac at the gender level. Blue = male, red = female. Analysis of the differentially abundant bacterial genera identified by **(D)** gender, **(E)** age, **(F)** BMI, and **(G)** physical activity. X-axis: evaluated demography, Y-axis: average abundance percentage. Alpha diversity for BMI using **(H)** Shannon’s index **(I)** observed_OTUs **(J)** faith_pd. Y-axis: alpha diversity indices in the respective metrics.

Qiime2 plugin “feature-classifier classify-sklearn” was performed on the obtained feature tables using the Greengene classifier, and overall 18 bacterial phyla were detected. Major detected phyla were Firmicutes (48.2 ± 21.3%), Bacteroidetes (30.7 ± 19.7%), Proteobacteria (5.0 ± 8.1%), Actinobacteria (4.0 ± 6.1%), and Fusobacteria (0.8 ± 2.9%) (**Figure 1A**). At the genus level, *Bacteroides* (20.5 ± 16.7%), *Faecalbacterium* (4.2 ± 4.1%)*, Prevotella* (4.4 ± 9.7%)*, Blautia* (3.1 ± 4.38%), and *Phascolarotobacterium* (3.0 ± 5.0%) were the most common (**Figure 1B**).

#### 3.1.2 Microbial Community Structure

**Figures 1D–G** shows the average individual abundance of differentially abundant bacteria identified in various demographics. Genus *Megamonas* was highly abundant in males (p = 0.0498) and overweight people (p = 0.052) compared to females and normal weight people. Genus *Prevotella* was significantly abundant (p = 0.037) in middle aged (45 – 64 years) and old aged people (> 64 years) than adults (18 – 44 years). Physical activity and lifestyle i.e., sleeping habits, also aided in defining gut microbiome composition. Subjects performing high to moderate levels of exercise every week showed a significantly greater abundance (p = 0.044) of butyrate-producing *Faecalibacterium* than less active subjects. Genus *Alistipes* was present at significantly higher levels (p = 0.012) in candidates with abnormal lifestyle/irregular sleeping habits (details in item S1).

#### 3.1.3 Alpha and Beta Diversity

**Figure 1C** shows the beta diversity analysis of gender for the Taiwanese population. As expected, no distinct cluster formation was observed between any of the assessed parameters by the weighted UniFrac metric due to similarity in the geographical demographics and lifestyle. In terms of alpha diversity, Shannon’s index (p = 0.016), observed_otus index (p = 0.005), and Faith’s phylogenetic diversity (faith_PD) index (p = 0.009) showed significant differences between normal and overweight people, with normal weight people showing higher diversity than overweight people (**Figures 1H–J**). Other characteristics such as gender, age, physical activity, etc., showed insignificant differences on all these indices (details in item S1).

### 3.2 Comparison of Microbiome Composition in Healthy *vs.* HD and PD Patients

Multiple studies have shown the impact of changes in the gut microbiome on non-communicable diseases. With the high prevalence of CKD in Taiwan, further analysis was conducted to reveal if any correlation of CKD with the gut microbiome exists which might affect its progression. Also, as CKD patients are administered different types of dialysis treatments, it is relevant to check how the treatment types can affect bacterial composition. **Table 2** presents the demographic description of the CKD patients. A total of 38 dialysis patients (16 HD and 22 PD) were compared with a selected cohort of 26 normal control subjects (matched by age and gender) from the TMB healthy panel for conducting a case-control microbiome analysis.

#### 3.2.1 Bacterial Composition

**Figure 2:**
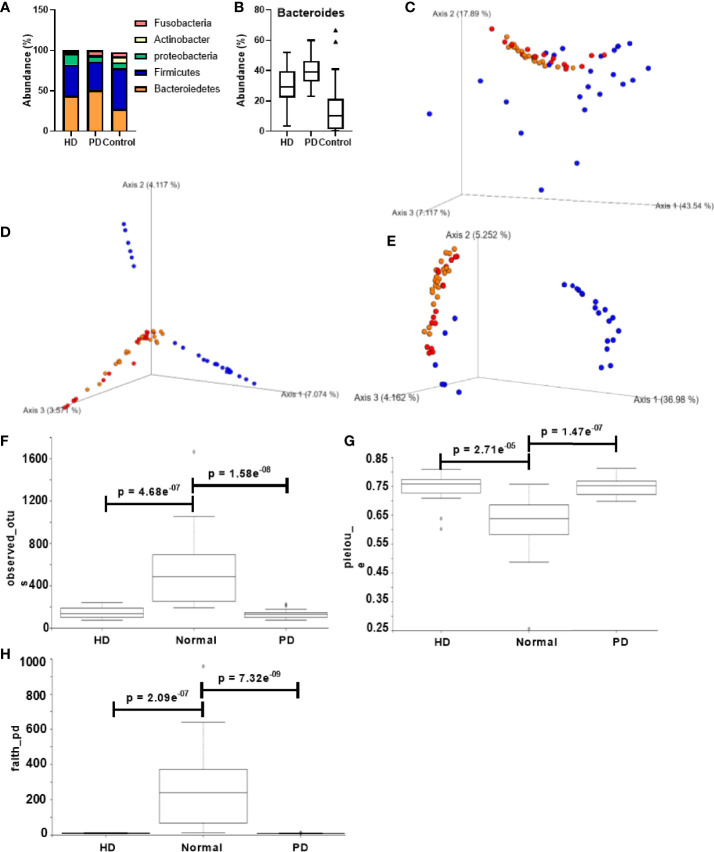
Comparison of gut microbiota composition in CKD patients and healthy control groups. **(A)** Taxonomic abundance at the phylum level for HD, PD, and normal groups. **(B)** Bacteroides abundance percentage compared across different groups. PCoA of the beta diversity values based on **(C)** weighted UniFrac, **(D)** Bray – Curtis, and **(E)** unweighted UniFrac. Yellow = PD, red = HD, blue = normal. Alpha diversity analysis HD, PD, and normal groups based on **(F)** observed_otus, **(G)** pielou_e, and **(H)** faith_pd. Y-axis: alpha diversity indices in the respective metrics.

Taxonomic analysis at the phylum level is shown in **Figure 2A**. The major components of the HD, PD, and control group were Bacteroidetes (43.9%, 50.6% and 25.6% respectively), Firmicutes (38.4%, 36.8% and 50.6% respectively), Proteobacteria (13.67%, 7.35% and 7.53% respectively), Actinobacter (2.198%, 0.79% and 7.04% respectively), and Fusobacteria (1.90%, 5.83%, 5.14%). The comparison of the overall microbiome composition between healthy controls and dialysis patients is shown in the supplementary data (**Figures S2A, B**). We observed that the dominant taxa changed from Firmicutes in control subjects to Bacteroidetes in HD and PD patients. At the genus level, *Bacteroides* was significantly more abundant in HD (p = 0.0013) and PD patients (p = 3.176e^-09^) than the control group (**Figure 2B**). Interestingly, the abundance of *Bacteroides* was significantly different between HD and PD patients (p = 0.0028), with higher abundance in PD patients (**Figure 2B**). Differences in distribution of age and other demographics may have partially attributed to this difference.

#### 3.2.2 Alpha and Beta Diversity

**Figures 2C–E** shows the alpha and beta diversity between healthy and dialysis (HD and PD) patients. Distinct clusters were formed for control *vs*. dialysis patients in all the metrics, suggesting a significant difference in microbiome composition. However, between HD and PD patients, no significant diversity was observed. Alpha diversity analysis between dialysis patients and the control group displayed Shannon’s diversity index of p = 0.052, faith_pd index of p = 7.13 e^-11^, Pielou’s evenness of p = 1.6 e^-08^ and observed OTUs index of p = 2.12 e^-10^ (**Figures S2C–F**). Individual analysis did not reveal any significant difference between HD, PD, and control subjects for Shannon’s metric. However, Pielou’s evenness, Faith’s phylogenetic diversity, and observed OTUs showed significant differences between both HD and control subjects (p = 2.71e^-05^, 2.09 e^-07^, and 4.68 e^-07^, respectively) and PD and control subjects (p = 1.47 e^-07^, 7.32 e^-09^, and 1.58 e^-08^, respectively) (**Figures 2F–H**). None of the metrics displayed any significant difference between HD and PD patients.

#### 3.2.3 Microbial Community Structure

**Figure 3:**
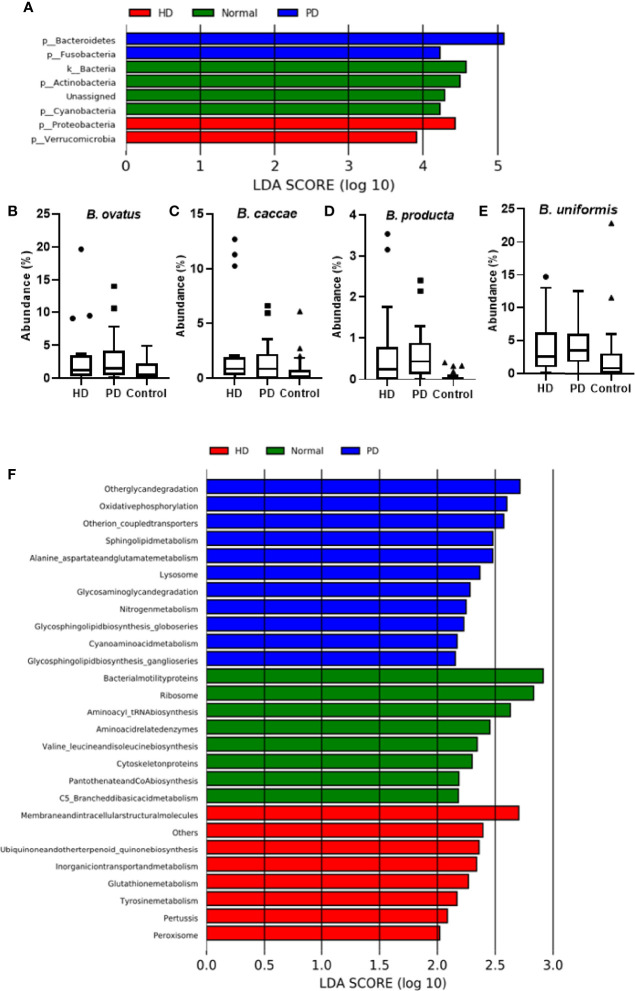
Analysis of Bacterial Community in Dialysis Patients. **(A)** LEfSe analysis of bacterial taxa at the phylum level for HD, PD, and control groups. Average abundance percentage of identified bacterial species between the three groups for **(B)** *B. ovatus*, **(C)** *B. caccae*, **(D)** *B. producta*, and **(E)** *B. uniformis*. **(F)** PICRUSt analysis showing functional profiles of the microbial samples from different groups at Kyoto Encyclopedia of Genes and Genomes (KEGG) level 3.

As shown by LEfSe (**Figure 3A**), phyla Bacteroidetes and Fusobacteria were identified to be relatively abundant in PD patients, whereas phyla Proteobacteria and Verrucomicrobia were predominant in HD patients. Several abundant bacterial species were identified in HD and PD patients (S3). *Bacteroides ovatus* was significantly abundant in HD (p = 0.03) and PD patients (p = 0.0033) when compared to the control group (**Figure 3B**), with average individual abundance in HD, PD, and control patients of 3.5 ± 5.2%, 3.0 ± 3.7%, and 1.1 ± 1.3%, respectively. Another abundant species identified in HD patients was *Bacteroides caccae* (p = 0.048), with an average abundance of 2.7 ± 4.4% (**Figure 3C**). There was no significant difference in abundance between PD patients and the control group (p = 0.35), although the average abundance was 1.5 ± 2.0% and 0.7 ± 1.3% in these groups, respectively. *Blautia producta* was the only species with a significantly higher presence (p = 0.02) in PD patients (20/22) than controls (15/26). The abundance was also significantly higher in PD patients (p = 4.48e^-06^) than controls (**Figure 3D**). In HD patients, although the difference in presence was insignificant in comparison to the control group, the abundance was significantly higher (p = 0.05) (**Figure 3D**). The average individual abundance of this bacterium in HD, PD, and control groups was 0.72 ± 1.1%, 0.6 ± 0.7%, and 0.06 ± 0.11%, respectively. *Bacteroides uniformis* was also significantly more abundant in HD patients (p = 0.008) and PD patients (p = 0.0002) than the control group (**Figure 3E**), with an average abundance of 4.2 ± 4.4%, 4.3 ± 3.4%, and 2.6 ± 4.9%, respectively.

#### 3.2.4 Predictive Functional Analysis

PICRUSt was used to predict the function of the most abundant gene sequences identified in the fecal microbiota, based on the constructed OTUs, according to the KEGG orthology database (**Figure 3F**). In PD patients, pathways such as “Other glycan degradation”, “Other ion coupled transporters”, and “Sphingolipid metabolism” were highly enriched, whereas in HD patients “Membrane and intracellular structural molecules”, “Ubiquinone and other terpenoid-quinone biosynthesis”, and “Inorganic ion transport and metabolism” pathways were enriched. In the healthy cohort, “Bacterial motility proteins”, “Ribososme”, and “Amino acyl_tRNA biosynthesis” pathways were highly functional.

## 4 Discussion

Characterizing the healthy gut microbiome is important for understanding the changes associated with diseases and tracking the relation of microbiota with the possible advent of or complications linked with disease. This pilot study created a healthy population gut microbiota reference panel for Taiwanese patients, TMB. The gut microbiome affects virtually all aspects of human health; therefore, understanding the underlying mechanisms and obtaining scientific evidence is crucial in unravelling the regulatory role of gut microbiota in the onset and progression of disease. Stability of a microbial community is perceived as a functional property indicating the healthy status of that community. Characterizing biomarkers related to disruption of a healthy microbiome, or unravelling biomarkers that make a healthy individual susceptible to infection or disease, is essential in understanding the role of the microbiome in human health and diseases.

In our study we identified several microbiota related to different demographics such as age, gender, BMI, physical activity, and sleeping habits in the Taiwanese population. We identified genus *Megamonas* significantly abundant in Taiwanese males. A previous study identified genus *Megamonas* as one of the highly abundant genera in healthy Japanese males (Takagi et al., 2019), whereas the same study and another Chinese study identified genus *Ruminococcus* as differentially abundant in females (Gao et al., 2018). Here, for healthy Taiwanese females, genus *Pyramidobacter* is being reported for the first time as significantly abundant.

Overweight (24 ≤ BMI < 27) Taiwanese people showed a significant abundance of genus *Megamonas*. Other Asian studies have also reported the abundance of *Megamonas* in obese people (BMI ≥ 27) (Chiu et al., 2014; Chen et al., 2020). In our study, we identified a significant abundance of this genus in overweight people as well. This suggests similarity of the gut microbiome among overweight and obese individuals of Asian origin, which can be attributed to their similar food habits. However, the specific regulatory mechanism of *Megamonas* is yet to be determined in the identified demographics. Also, we didn’t find any correlation between gender and bodyweight. Additionally, in normal weight healthy people, 7 relatively abundant genera (*Faecalibacterium, Roseburia, Blautia, Ruminococcus, Coprococcus, Dorea*, and *Peptoniphilus)* were identified (**Figure S1C**). All of the mentioned genera (except *Peptoniphilus*) have been reported in prior Chinese studies. (Zhang et al., 2015; Zhang et al., 2019). These results suggest that similar food habits could be a major driving factor in comparable gut microbiome composition of Chinese and Taiwanese people.

We identified genus *Prevotella* as highly abundant in the middle aged and adult Taiwanese population. This finding is consistent with another Chinese study showing abundance of genus *Prevotella* in the 60 – 79 age group (Bian et al., 2017.) Another study identified *Prevotella* as commonly dominant in adult gut samples from native or rural villages of Burkina-Faso, South Africa, Venezuela, Malawi, and Russia (Gorvitovskaia et al., 2016). This suggests the commonality of the gut microbiome pertaining to certain age group.

People performing high to moderate exercise on a regular basis showed significantly higher abundance of genus *Faecalibacterium* and of species *F. prausnitzii.* *Faecalibacterium* species, especially *F. prausnitzii*, are well-known butyrate-producing bacteria in the human gut (Parada Venegas et al., 2019) and are important in preventing colorectal cancer; they also are useful as protective agents in hemoglobinopathies, genetic metabolic diseases, hypercholesterolemia, insulin resistance, and ischemic stroke (Canani et al., 2011). Bressa et al. showed an increase in *F. prausnitzii* in women performing moderate workouts compared to sedentary women. (Bressa et al., 2017) Another study assessed the microbiota of the American Gut Project and concluded that increasing exercise frequency from never to daily showed a higher abundance of *F. prausnitzii*, which contributes to a healthier gut environment. (McFadzean, 2014) These results suggest the role of exercise in enhancing gut efficiency irrespective of geographical differences.

Genus *Alistipes* was present to a significantly greater degree in people with abnormal sleeping habits compared to those with normal sleeping habits. Jiang et al. have reported a higher abundance of this genus in major depressive disorder patients in the Chinese population (Jiang et al., 2015). A Norwegian study also reported the abundance of *Alistipes* in depression patients compared to healthy controls (Naseribafrouei et al., 2014). *Alistipes* is an indole-positive genus and could influence the availability of the serotonin precursor, tryptophan (Jiang et al., 2015). Since multiple studies have related sleep disorders with depression (Scott et al., 2017), our finding further supports the role of regular sleep habits in promoting mental health.

Gut dysbiosis has been identified as a primary factor in regulating the toxins responsible for further complications in CKD patients. The microbiota are solely responsible for the generation of indoxyl sulfate (IS) (Carter et al., 1966; Wikoff et al., 2009), one of the important uremic toxins circulating in the body in cases of impaired kidney function. In animal models, IS has been shown to accelerate tubular cell injury and induce interstitial fibrosis and glomerular sclerosis (Niwa and Ise, 1994; Niwa et al., 1994). Also, IS helps in triggering vascular dysfunction and aortic calcification in CKD patients, leading to cardiovascular mortality (Adijiang et al., 2008; Niwa, 2010). *Bacteroides* species are capable of producing indole, (Niwa et al., 1994) which is then hydroxylated to indoxyl (Banoglu et al., 2001) and eventually is converted to IS(Banoglu and King, 2002). Tryptophanases (Tnases) are the enzymes (present only in bacteria) responsible for conversion of tryptophan to indole, pyruvate, and ammonia (Newton and Snell, 1964). Devlin et al. computationally identified a majority of the Tnase genes in *Bacteroides* (Devlin et al., 2016). They also showed a reduction in IS levels in a conventional mouse when colonized with Tnase-deficient *Bacteroides*, conclusively proving the role of Tnase in production of uremic toxins (Devlin et al., 2016).

Therefore, in this study, we compared the gut microbiome of CKD dialysis patients with a control group (TMB) and identified several important bacterial taxa (S3). Our study concurs with the above explanations and another Chinese study identifying abundance of the genus *Bacteroides* in dialysis patients (Jiang et al., 2017). At the species level, we identified *B. caccae* and *B. ovatus* as significantly abundant in dialysis patients compared to the control group (**Figures 3B, C**, **S3**). Specifically, both species were relatively abundant in HD patients. In a recent study, the authors isolated multiple bacterial strains from CKD patients to characterize their ability to produce protein-bound uremic toxins (PBUT) above the limit of detection (Gryp et al., 2020). They showed *B. caccae* capable of producing *p*-cresol, phenol, and indole-3-acetic acid (IAA) in both aerobic and anaerobic conditions and *B. ovatus* could produce phenol and IAA in anaerobic conditions (Gryp et al., 2020). We also identified a relative abundance of *B. uniformis* in HD patients, which has been characterized as one of the few bacterial species capable of producing all three PBUTs in anaerobic conditions, as previously reported (Gryp et al., 2020). *Ruminococcus gnavus* and *Ruminococcus torques* were other differentially abundant bacteria observed in dialysis patients, specifically in HD patients (S3). Lun et al. had identified these bacteria as specific biomarkers in kidney disease patients in China (Lun et al., 2018). Another study identified increases in *Ruminococcus* species in patients with end stage renal disease (Wang et al., 2020). Another Taiwanese study identified the abundance of genera *Streptococcus, Parabacteroides*, and *Dorea* in HD patients; however, species level identification was not done (Yang et al., 2021).

In PD patients, we identified *B. producta* as significantly more abundant than in the control group. Previous studies have shown the abundance of genus *Blautia* in Taiwanese CKD patients (Wu et al., 2020); however, for the first time species level identification *via* 16S rRNA sequencing has been done for this population. Another study reported the enrichment of genus *Blautia* in HD patients (Stadlbauer et al., 2017). Studies with mouse models have shown a decrease in *B. producta* in mouse gut microbiota after nephrectomy, proving the association of this bacteria with CKD. (Chaves et al., 2018) Another animal study identified enrichment of the *Blautia* genus in rats with unilateral ureteral obstruction (Chen et al., 2019), which is a general cause of renal impairment and can lead to tubular atrophy, interstitial fibrosis, inflammation, and eventually irreversible renal injury. (Better et al., 1973; Sacks et al., 1989).

In PD patients, “nitrogen metabolism” was one of the highly functional pathways. Urea is the end product of nitrogen metabolism and is present in higher concentrations in patients with renal failure (Vanholder et al., 2018). High functional abundance of nitrogen metabolism is a precursor to higher concentrations of urea in PD patients. In HD patients, “tyrosine metabolism” was shown to be highly functional. Tyrosine is one of the aromatic amino acids whose metabolization is positively related to higher uremic toxin production (Dodd et al., 2017). Thus, these pathways are directly related to the production of circulating uremic toxins in CKD patients, which plays a functional role in the further deterioration of their condition.

In conclusion, we have analyzed a healthy reference panel called Taiwan Microbiome Baseline and identified many bacterial taxa related to specific demographics. Using this panel as control group, we identified many unique bacterial markers at the species level for kidney disease patients from Taiwan. These species could be principal factors in further aggravating the disease or its complications. We identified clear demarcations in bacterial diversity in the beta diversity analysis and identified the driving bacterial taxa for healthy, HD, and PD groups. As these were only first- or second-year dialysis patients, long-term analysis of CKD patients during treatment is proposed to monitor the microbiome distribution and related changes. Since many studies have attributed bacterial metabolites and host-bacteria interactions to life-threatening diseases, identifying the pathogenic bacteria in our case could open new vistas for effective bacterial therapy in patients with renal impairment. The bacterial markers we identified in CKD patients, and their functional pathways, are worth investigating in the future to determine their potential as causal agents of further complications in CKD. Moreover, the bacterial markers identified here are different from an Austrian study of gut dysbiosis in CKD patients (Stadlbauer et al., 2017), fortifying the importance of geography and diet in modulating the gut microbiota. Hence, to target gut microbiota for diagnostic or therapeutic purposes, identifying population specific gut dysbiosis pertaining to the disease is an important factor.

## 5 Limitations

Our study has several limitations including diet control of the healthy subjects. Also, the blood markers associated with CKD were not correlated with the identified OTUs to conclusively proof the role of identified bacteria in aggravating the conditions of the CKD patients.

## Data Availability Statement

The original contributions presented in the study are included in the article/**Supplementary Material**, further inquiries can be directed to the corresponding author/s.

## Ethics Statement

The studies involving human participants were reviewed and approved by Institutional Review Board of the National Taiwan University (201801085RINB) National Cheng Kung University Hospital (B-ER-106-058). The patients/participants provided their written informed consent to participate in this study.

## Author Contributions

EC, C-YK, T-PL, AC, and M-CW conceptualized and designed the study. SS performed all data analysis. SS and AC wrote the manuscript. W-HL, C-HT, J-JW, M-HT, and M-CW contributed to the collection of samples. M-HT, J-WC, I-HH, and Y-HH extracted the DNA and performed NGS assay. AC, SS, EC, C-YK, L-CL, M-HT, and T-PL were responsible for manuscript revision. EC, C-YK, M-CW, L-CL, M-HT, and T-PL managed and coordinated the project. All authors contributed to the article and approved the submitted version.

## Funding

This work was supported in part by the Ministry of Science and Technology (MOST 110-2634-F-002 -044 -, NTU Centers of Genomic and Precision Medicine, MOST 110-2321-B-002 -015 –, MOST 110-2320-B-A49A-524 – and MOST 110-2323-B-A49A-524-) and the Yen Tjing Ling Medical Foundation, Taiwan (CI-110-13).

## Conflict of Interest

The authors declare that the research was conducted in the absence of any commercial or financial relationships that could be construed as a potential conflict of interest.

## Publisher’s Note

All claims expressed in this article are solely those of the authors and do not necessarily represent those of their affiliated organizations, or those of the publisher, the editors and the reviewers. Any product that may be evaluated in this article, or claim that may be made by its manufacturer, is not guaranteed or endorsed by the publisher.
