# Supplementary material for: Uremic Toxin-Producing Bacteroides Species Prevail in the Gut Microbiota of Taiwanese CKD Patients: An Analysis Using the New Taiwan Microbiome Baseline
Source: Front Cell Infect Microbiol. 2022 Apr 26;12:726256. doi: 10.3389/fcimb.2022.726256 (PMC9086402; doi:10.3389/fcimb.2022.726256)
Supplement: Supplementary file 1 [file DataSheet_1.zip › S1_Supplementary_TMB_results.docx]

**Taiwan Microbiome Baseline – Results**

Figures S1 ((A) – (F)) shows the differentially abundant taxa identified by LefSe in different demographics. We performed further statistical analysis such as Fisher’s exact test and Wilcoxon test to identify the significant biomarkers.

Figure 1(D) displays the differentially abundant genera based on gender, where *Megamonas* was identified in 63.1% of males (24/38) and 51.8 % (42/81) of females with significant abundance level in males (p = 0.0498). Average abundance in males is 8.5 ± 18.8 % whereas in females it is 1.0 ± 2.2 %. Genus *Pyramidobacter* was identified in 12.34% (10/81) females and none of the males (p = 0.029).

Age proved to be another factor modulating the abundance of gut microbiomes (Figure 1(E)). Genus *Prevotella* was identified almost uniformly in all subjects i.e., 87.2% in adults (18 – 44 years), 86.7% in middle aged (45 – 64 years) and 84.2% in old aged (> 64 years) subjects. However, the abundance was significant (p = 0.037) in middle aged and old aged people with average abundance at 6.3 ± 11.7 % and 9.2 ± 11.4 %, respectively than adults with an average abundance level of only 2.9 ± 8.2 %.

BMI was also one of the drivers in characterizing the bacterial community (Figure 1 (F)). *Megamonas* was identified in 65.78% (25/38) of overweight people and 50.61% (41/81) of normal weight people. The abundance of genus Megamonas was significantly higher in overweight people (p = 0.052) with an average abundance of 7.4 ± 18.14 % whereas for normal weight people, the average abundance was only 1.52 ± 4.34 %.

Physical activity also influenced the gut microbiota composition (fig. 1 (G)). Candidates performing high to moderate exercises every week showed significant abundance (p = 0.044) in butyrate producing *Faecalibacterium* than other candidates. The average abundance in the former group was calculated as 4.50 ± 2.9 % while for latter it was 4.02 ± 5.03 %. Species level analysis in LefSe showed higher abundance of *F. prausnitzii*, a well-known butyrate producing, anti-inflammatory commensal bacteria, in candidates with high-moderate physical activity (Fig S1 (E)).

Another important factor for gut microbiome modulation was sleeping habits. Genus *Alistipes* showed significant presence (p = 0.014) in 54.3 % subjects with abnormal lifestyle (38/70) i.e., irregular sleeping habits than subjects with normal lifestyle subjects (15/49). Although, the abundance was significant (p = 0.012) in normal sleeping habits group averaging at 0.34 ± 0.94 % compared to abnormal sleeping habits population 0.17 ± 0.37 % (Fig. S1 (K)).

*Functional Prediction*

PICRUSt was used to predict the distinct functional pathways attributed to the studied parameters. Age, BMI, gender, physical activity and lifestyle showed varied pathway prediction for each category (Fig. S1 (G) – (J)). Level-3 of KEGG analysis showed highly enabled “Pentose and glucoronate interconversions” pathway in adults (18 – 44 years) (LDA > 2) whereas for older people (> 64 years), “Bacterial motility proteins” and “Bacterial chemotaxis” pathway was differentially enriched. Normal weight people showed more active “Inorganic ion transport and metabolism” pathway while overweight people showed elevated levels of “DNA repair and recombination proteins”, “Replication recombination and repair proteins”, “Aminoacyl tRNA biosynthesis” and “amino acid related enzymes” pathways.

“DNA repair and recombination proteins”, “Aminoacyl tRNA biosynthesis” and “Amino acid related enzymes” pathways were more enriched in males whereas in females “two-component system”, “inorganic ion transport and metabolism”, “Pentose and glucoronate interconversions” and “Glycoxylate and dicarboxylate metabolism” were significantly elevated.

Pathways such as “DNA repair and recombination proteins” and “pyrimidine metabolism” were enriched in high to moderate exercise performing candidates whereas “Bacterial secretion system” and “Inorganic ion transport and metabolism” pathway was enriched in other candidates.

No differentially enriched pathways were shown for lifestyle demography.

S1. Healthy Taiwan Microbiome Baseline (TMB) analysis. Linear Discriminant Analysis (LDA) Effect Size (LefSe) analysis of bacterial taxa at (A) genus level for age group (b) genus level for gender (C) species level for BMI (D) genus level for physical activity (E) species level for physical exercise (F) genus level for lifestyle

Phylogenetic Investigation of Communities by Reconstruction of Unobserved States (PICRUSt) analysis showing functional profiles of the microbial samples at Kyoto Encyclopedia of Genes and Genomes (KEGG) level 3 for (G) age group (H) BMI (I) physical activity (J) gender

(K) Average abundance percentage of Genus *Alistipes* between normal and abnormal lifestyle group. x-axis: normal and abnormal lifestyle group, y-axis: abundance percentage. p-value: 0.012


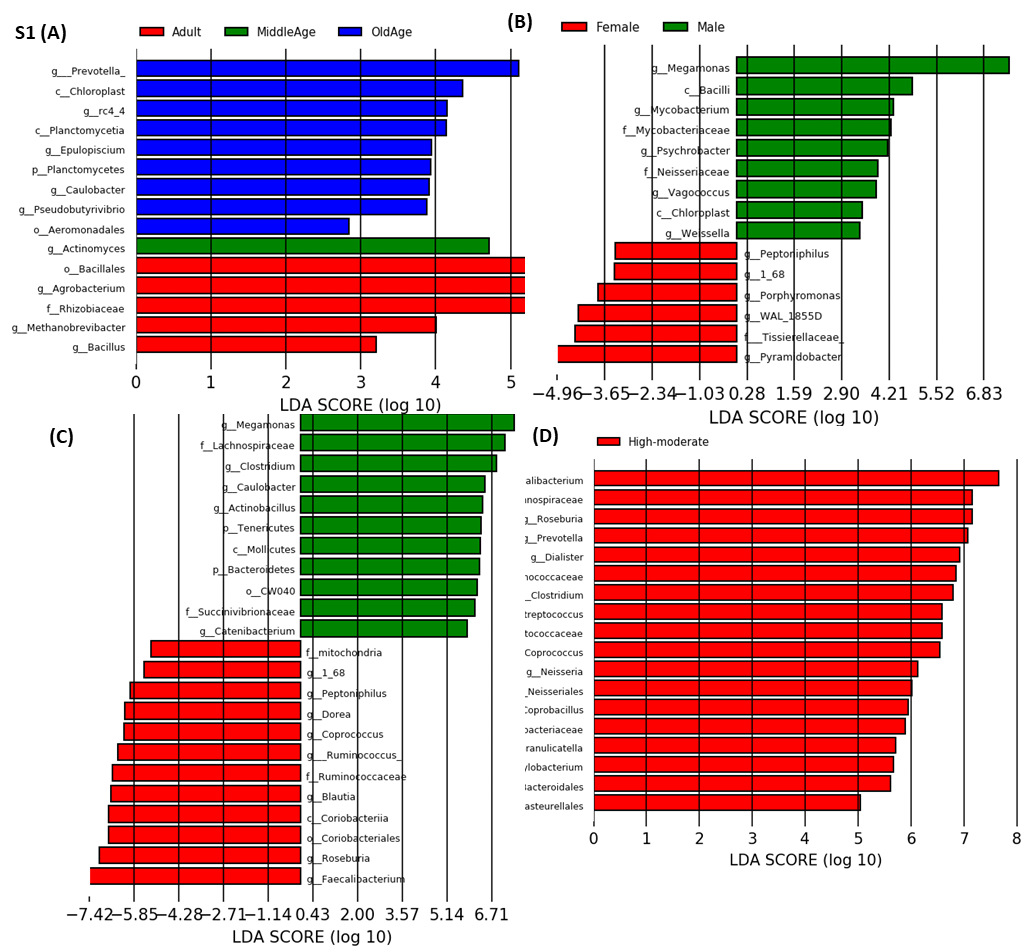


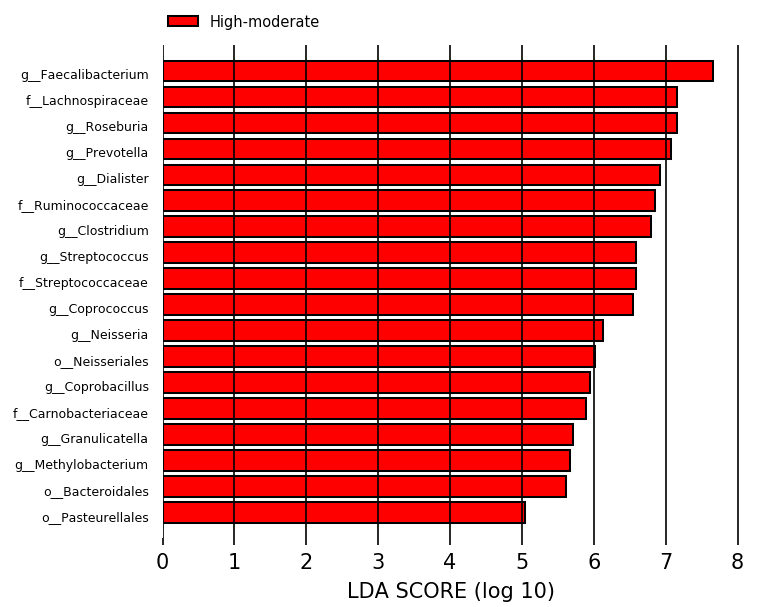

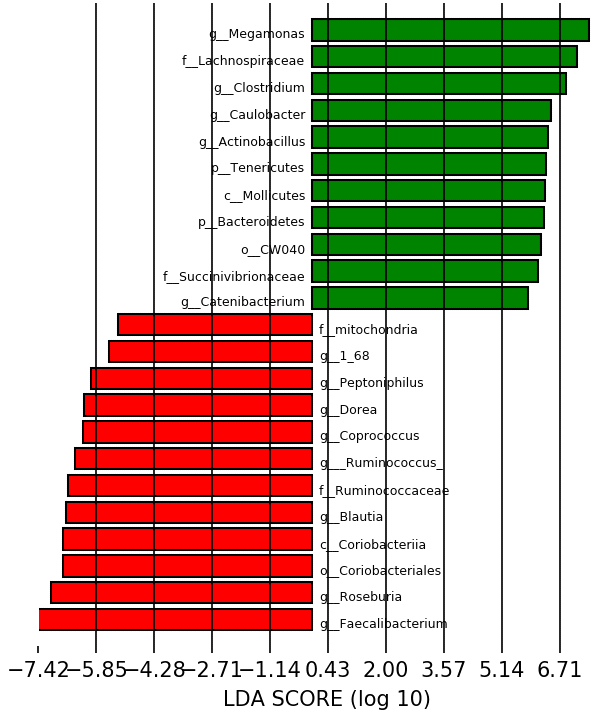

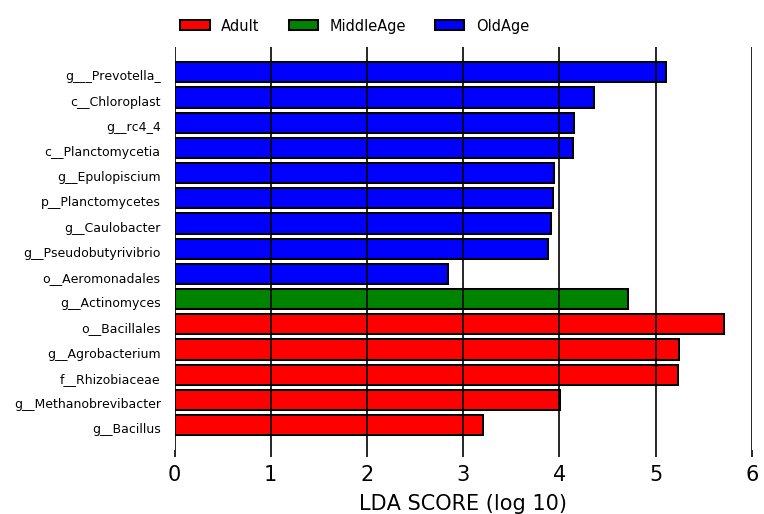

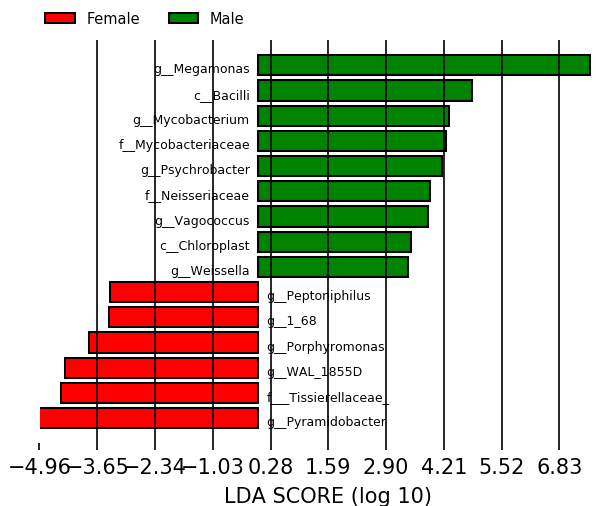


**S1 (A)**

**(B)**

**(C)**

**(D)**


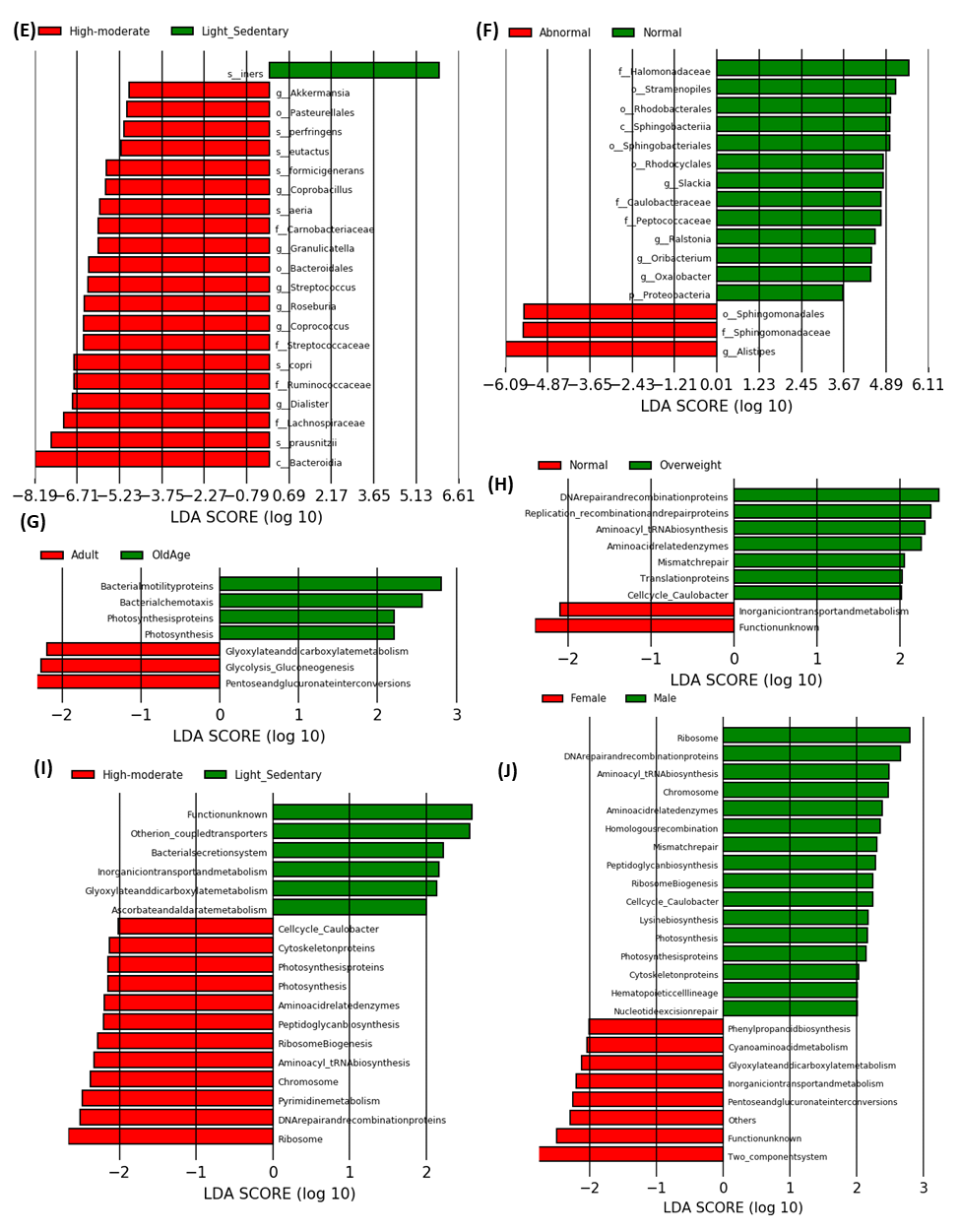


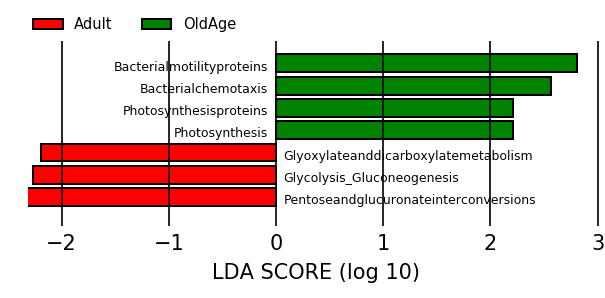

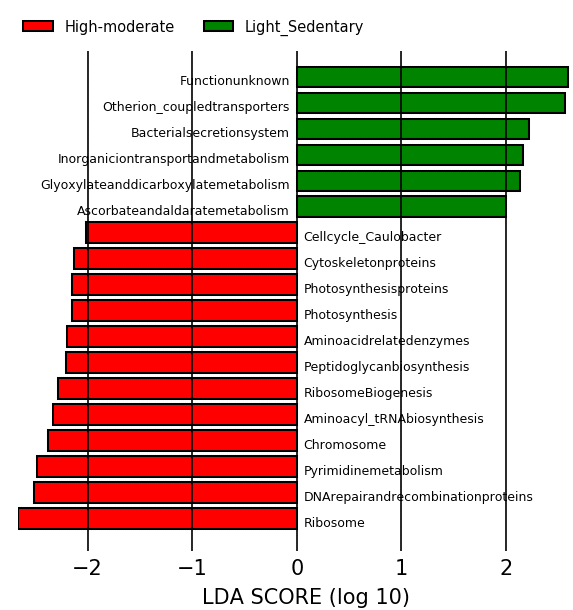

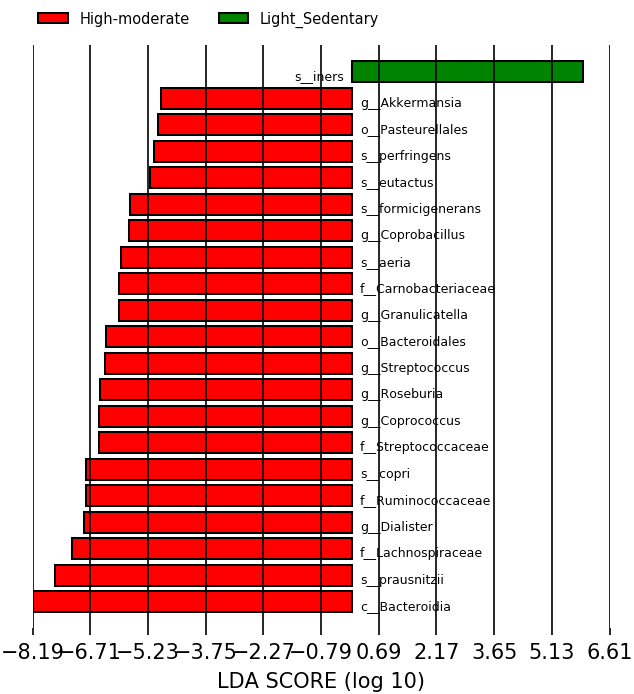

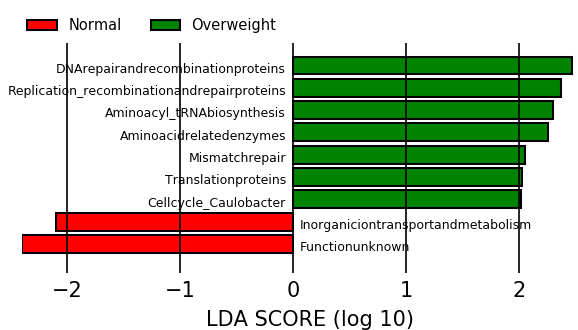


**(E)**

**(G)**

**(H)**

**(I)**

**(J)**


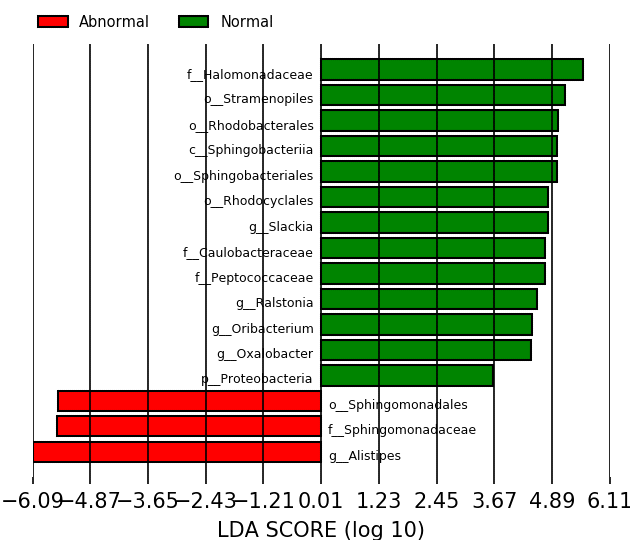

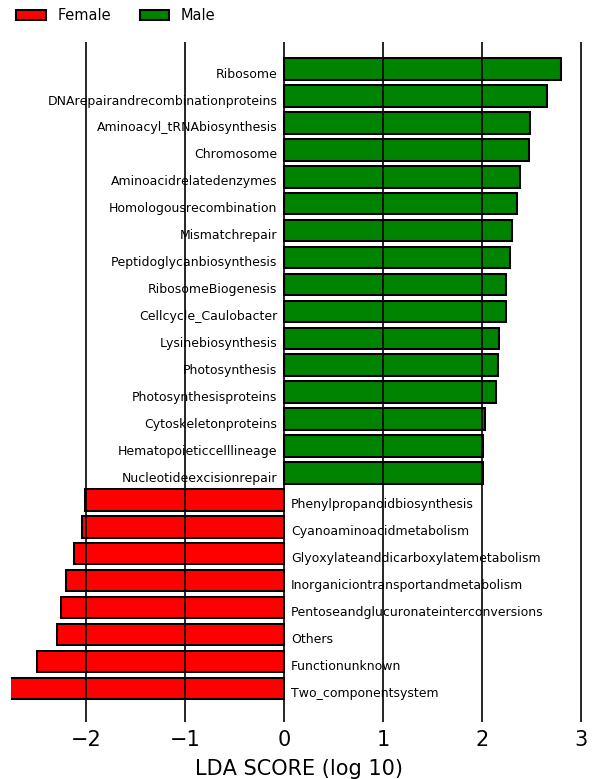


**(F)**


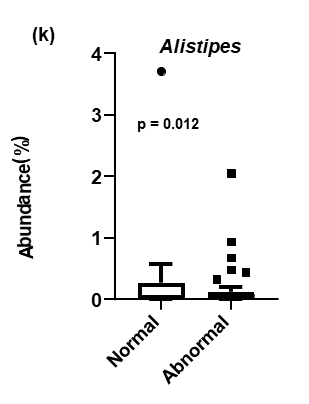


**(k)**

**N**

**o**

**r**

**m**

**a**

**l**

**A**

**b**

**n**

**o**

**r**

**m**

**a**

**l**

**0**

**1**

**2**

**3**

**4**

***Alistipes***

**A**

**b**

**u**

**n**

**d**

**a**

**n**

**c**

**e**

**(**

**%**

**)**

**p = 0.012**
