# Supplementary material for: Uremic Toxin-Producing Bacteroides Species Prevail in the Gut Microbiota of Taiwanese CKD Patients: An Analysis Using the New Taiwan Microbiome Baseline
Source: Front Cell Infect Microbiol. 2022 Apr 26;12:726256. doi: 10.3389/fcimb.2022.726256 (PMC9086402; doi:10.3389/fcimb.2022.726256)
Supplement: Supplementary file 1 [file DataSheet_1.zip › S2_Supplementary_TMB_CKD.docx]

Analysis of dysbiotic gut microbiota in Taiwanese dialysis patients by comparing with a new healthy Taiwanese Microbiome Baseline

S2. Dialysis patients vs healthy control group analysis.

Taxonomic classification at the (A) phylum and (B) genus level. x-axis: individual samples, y-axis: percentage of bacteria in individual samples. Alpha diversity for dialysis patients and healthy controls calculated as per (A) Shannon’s index (B) faith_pd (C) pielou_e (D) observed_otus metrics. x-axis: dialysis and healthy groups, y-axis: alpha diversity indices in respective metrics
