# Supplementary figures and images for: Uremic Toxin-Producing Bacteroides Species Prevail in the Gut Microbiota of Taiwanese CKD Patients: An Analysis Using the New Taiwan Microbiome Baseline
Source: Front Cell Infect Microbiol. 2022 Apr 26;12:726256. doi: 10.3389/fcimb.2022.726256 (PMC9086402; doi:10.3389/fcimb.2022.726256)

HD Normal PD

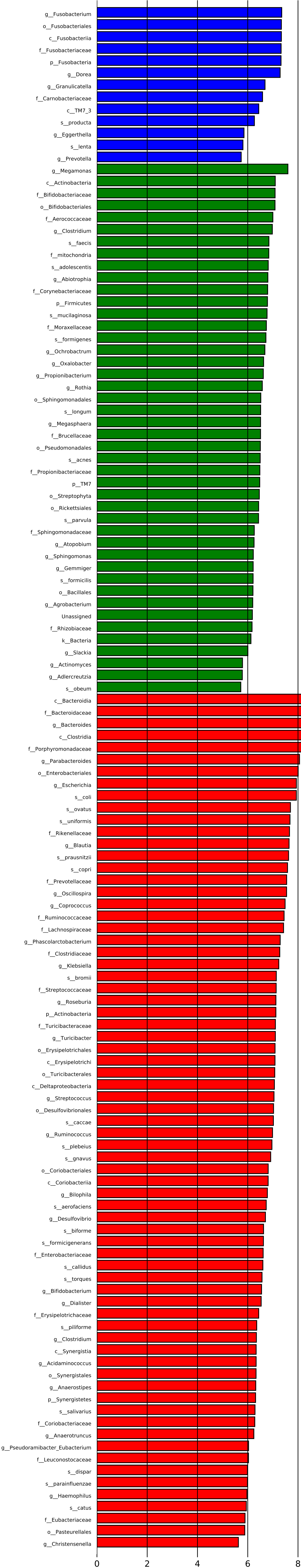

Supplement: Supplementary file 1 [file DataSheet_1.zip › S3-gut_microbiota_TMB_and_CKD_patients.pdf]
